# Flavonoids and Other Compounds from *Ouratea ferruginea* (Ochnaceae) as Anticancer and Chemopreventive Agents

**DOI:** 10.3390/molecules17077989

**Published:** 2012-07-03

**Authors:** Queli C. Fidelis, Rosane N. Castro, Giselle M. S. P. Guilhon, Silvane T. Rodrigues, Cristiane M. C. de Salles, João B. de Salles, Mario G. de Carvalho

**Affiliations:** 1 Programa de Pós-Graduação em Química, ICE-Universidade Federal Rural do Rio de Janeiro, 23890-000, Seropédica, RJ, Brazil; Email: nora@ufrrj.br (R.N.C.); salles.cristiane@gmail.com (C.M.C.S.); 2 Faculdade de Química, Instituto de Ciências Exatas e Naturais, Universidade Federal do Pará, Av. Augusto Corrêa 01, 66075-110, Belém, PA, Brazil; Email: giselle@ufpa.br; 3 Departamento de Botânica, Embrapa Amazônia Oriental, Tv. Enéas Pinheiro s/n, 66077-530, Belém, PA, Brazil; Email: comus@cpatu.embrapa.br; 4 Laboratório de Bioquímica, Centro de Ciências Biológicas e da Saúde, Centro Universitário da Zona Oeste (UEZO), 23070-200, Rio de Janeiro, RJ, Brazil; Email: cotbcb@uezo.rj.gov.br; 5 Núcleo de Pesquisa de Produtos Naturais, Bloco H, CCS-UFRJ, Ilha do Fundão, 21941-970, Rio de Janeiro, RJ, Brasil; Email: mgeraldo@ufrrj.br

**Keywords:** *Ouratea ferruginea*, biflavonoids, isoflavones, chemopreventive agents

## Abstract

The chemical study of the extracts from leaves and stems of *Ouratea ferruginea* allowed the identification of a new isoflavone, 5-hydroxy-7,3′4′5′-tetramethoxyisoflavone, and twenty two known compounds, including friedelin, 3β-friedelinol, lupeone, a mixture of sitosterol, stigmasterol and campesterol, sitosteryl- and stigmasteryl-3-*O*-β-D-glucopyranosides, 5,4′-dihydroxy-7,5′,3′-trimethoxyisoflavone, 5,4′-dihydroxy-7,3′-di-methoxyisoflavone (7,3′-di-*O*-methylorobol), 5,7,4′-trihydroxy-3′,5′-dimethoxyisoflavone (piscigenin), 2*R*,3*R*-epicatechin, syringic acid, 2,6-dimethoxybenzoquinone, 2,6-dimethoxyhydroquinone, syringic and ferulic aldehyde, a mixture of vanillic acid, 1-hydroxy-2-methoxy-4-(1*E*-3-hydroxy-1-propenyl)-benzene and 3,5-dimethoxy-4-hydroxy-dihydrocinamaldehyde, besides amenthoflavone and 7-*O*-methylamenthoflavone (sequoiaflavone) which are considered as chemotaxonomic markers of *Ouratea*. The structures were identified by IR, ^1^H- and ^13^C-NMR and GC-MS, HPLC-MS, besides comparison with literature data. The inhibitory effects of 5,4′-dihydroxy-7,5′,3′-trimethoxyisoflavone, 7,3′-di-*O*-methylorobol, piscigenin and 7-*O*-methylamenthoflavone on cytochrome P450-dependent 7-ethoxycoumarin *O*-deethylase (ECOD) and glutathione *S*-transferase (GST) were evaluated *in vitro*. The 5,4′-dihydroxy-7,5′,3′-trimethoxy-isoflavone was the best inhibitor, inhibiting almost 75% of GST activity. Sequoiaflavone was the most potent inhibitor, inhibiting ECOD assay in 75%. These activities allow us to consider both these flavonoids as potential anticancer and chemopreventive agents.

## 1. Introduction

*Ouratea* Alb. genus is the largest within the Ochnaceae family with approximately 120 species [1]. Plants in this family are known to be rich in flavonoids and biflavonoids. Biflavonoids can be used as chemotaxonomic markers of the genus [2]. Some biflavonoids, as well as extracts of *Ouratea* species showed important biological activities such as DNA topoisomerase inhibition, cytotoxic, antitumoral activities and other pharmacological activities [3].

The cancer protective effects of flavonoids have been attributed to a wide variety of mechanisms, including free radical scavenging, modifying enzymes that activate or detoxify carcinogens, and inhibiting the induction of the transcription factor activator protein-1 (AP-1) activity by tumor promoters [4]. Among the enzymes, which are likely to play important roles in carcinogenesis that interact with flavonoids, cytochromes P450 (CYPs) and glutathione *S*-transferases (GST), are the most extensively studied.

Cytochrome P450s (monooxygenase enzymes) are enzymes that catalyze a variety of chemical reactions such as *O*-dealkylation, *N*-dealkylation, *O*-hydroxylation and *N*-hydroxylation and thus play an important role primarily in phase I of xenobiotic metabolism. They help their excretion and activate the endogenous compounds (estradiol, testosterone, 11-deoxycortisol, arachidonic acid and vitamin D) to modify the products that are involved in the regulation of physiological and cellular processes (homeostasis, endocrine control, inflammation and cellular proliferation) [5]. Many naturally occurring flavonoids have been shown to modulate the CYP450 system, including the induction of specific CYP isoenzymes, and the activation or inhibition of these enzymes.

Glutathione *S*-transferases (GSTs, EC 2.5.1.18) catalyze the binding of a large variety of electrophiles to the sulfydryl group of glutathione, that are involved in the detoxification of (oxygen) radicals, and have a main function in the binding and transport of a wide variety of harmful compounds [6]. The major carcinogenic agents are exogenous or metabolically generated reactive oxygen species (ROS) and electrophiles arising from the environment and from in vivo normal oxidative processes. Enhanced ROS levels are involved in initiation and promotion of tumor and may ultimately lead to carcinogenesis [7].

The present paper reports the first phytochemical study of extracts from stems and leaves of *Ouratea ferruginea* Engl, describing the isolation and identification of twenty four compounds, including five isoflavones and two biflavonoids. Four isolated compounds were evaluated on ECOD (CYP1A1) and GST *in vitro* inhibition.

## 2. Results and Discussion

**Figure 1:**
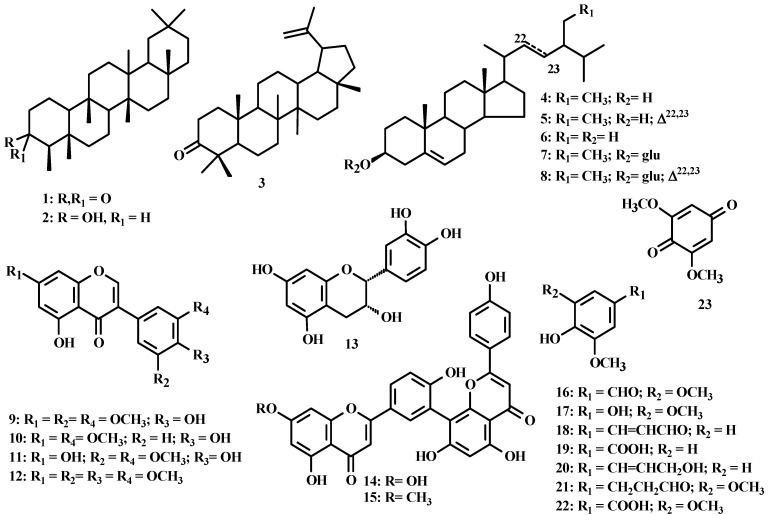
Structures of compounds identified in *Ouratea ferruginea*.

The chromatographic procedure with the extracts of *Ouratea ferruginea* afforded twenty three known compounds. Friedelin (**1**) [8], 3β-friedelinol (**2**) [9], a mixture of sitosterol (**4**), stigmasterol (**5**) and campesterol (**6**) [10], a mixture of sitosteryl-3-*O*-β-D-glucopyranoside (**7**) and stigmasteryl-3-*O*-β-D-glucopyranosides (**8**) [10], 5,4′-dihydroxy-7,5′,3′-trimethoxyisoflavone (**9**) [11], 7,3′-di-*O*-methylorobol (**10**) [12], piscigenin (**11**) [13], a mixture of 5-hydroxy-7,3′,4′,5′-tetramethoxyisoflavone (**12**) and (**9**), syringic aldehyde (**16**) [14], 2,6-dimethoxyhydroquinone (**17**) [15], ferulic aldehyde (**18**) [16], the mixture of vanillic acid (**19**), 1-hydroxy-2-methoxy-4-(1*E*-3-hydroxypropenyl)benzene (**20**) and 3,5-dimethoxy-4-hydroxydihydrocinamaldehyde (**21**) and 2,6-dimethoxybenzoquinone (**23**) [15], were identified from the stems extracts and a mixture of triterpenes, friedelin (**1**), 3β-friedelinol (**2**) and lupeone (**3**) [8], the steroids mixture sitosterol (**4**), stigmasterol (**5**) and campesterol (**6**) [10], 2*R*,3*R*-epicatechin (**13**) [17], amenthoflavone (**14**) [18], sequoiaflavone (**15**) [18] and syringic acid (**22**), from the leaves extracts. The structures of the compounds were proposed by ^1^H- and ^13^C-NMR and CD spectral data analysis and comparison with the literature data [8,9,10,11,12,13,14,15,16,17,18], including HPLC and GC-MS analysis. The structures are shown in Figure 1.

Compound **12** was obtained as a pale yellow precipitate. The ^1^H-NMR spectrum indicated the presence of a signal at *δ* 7.89 characteristic of H-2 of the heterocyclic ring of isoflavones, and a signal at *δ* 6.72 attributed to two chemically equivalent protons in the aromatic system H-2′ and 6′ of ring B, two doublets at *δ* 6.39 (1H, d, *J* = 2.5 Hz, H-6) and *δ* 6.41 (1H, d, *J* = 2.5 Hz, H-8) of the ring A; a chelated hydroxyl group *δ* 12.84 (1H, s, 5-OH) and four methoxyl groups at *δ* 3.87–3.89 (s, 3H each). The ^13^C-NMR spectrum indicated the presence of signals at *δ* 56.3 (C-3′, 5′), *δ* 60.9 (C-4′) and *δ* 55.9 (C-7), corresponding to the carbons of methoxyl groups attached to an aromatic ring. The carbonyl carbon was observed at *δ* 180.3 (C-4) and a signal of CH at *δ* 152.7 attributed C-2 of the heterocyclic ring of isoflavones. Further confirmation was obtained from the HMBC correlations: The proton H-2′,6′ (δ 6.72) correlated with C-1′ (*δ* 123.7), C-3′,5′ (*δ* 153.0), and C-4′ (*δ* 138.0). The compound **12** was identified by GC-MS analysis in the mixture of **9**. Both compounds showed retention time at Tr = 27.149; *m/z* = 358 (**12**) and Tr = 29.619; *m/z* = 344 (**9**) that are in agreement the proposed structures. The peaks *m/z* 167 (trimethoxyphenyl ion) detected in the mass spectrum of **12** confirmed the tetramethoxyisoflavone structure. This represents the first record of isoflavone **12** in the literature. The ^1^H- and ^13^C-NMR data of both pure compounds **9** and **12** (obtained by the treatment of **11** with diazomethane) are described in the Experimental.

### 2.1. Biological Activity

The tested flavonoids revealed important anti-cancer properties. These properties may be attributed to their capacity of inhibiting phase I enzymes or activating phase II enzymes. These phase I and phase II metabolizing enzymes are the key to carcinogen metabolism. Thus inhibition or enhancement of their activities are a significantly chemopreventative cancer effect. The potential of compounds listed in Table 1 to modulate the activity of drug-metabolizing enzymes, *i.e.*, phase I, cytochromes P450 (Cyp) enzymes and phase II detoxifying enzymes (gluthation *S*-transferase) was investigated.

**Table 1:** Activities values (%) of CYP-catalyzed ethoxycumarin *O*-desalkylation (ECOD) and gluthation *S*-transferase (GST) inhibited by flavonoid.

Compounds [0.08 mg/mL]	ECOD activity	GST activity
5,4′-OH-7,3′,5′-OMe-isoflavone**(9)**	66.9 ±27.7	25.3 ±5.3
7,3′-Dimethoxy-5,4′-dihydroxyisoflavone **(10)**	77.8 ± 11.6	ND *
5,7,4′-Trihydroxy-3′,5′-dimethoxyisoflavone **(11)**	53.5 ± 12.4	32.2 ±1.3
Sequoiaflavone **(15)**	24.8 ± 1.2	77.4 ± 6.7

* ND: Not detected. (%) Activity relative to control.

ECOD assay was selected as a phase I marker enzyme. ECOD is catalyzed by many CYP enzymes, including CYP1A1, CYP2A6, CYP2B6, and CYP2E1, and also CYP3A to a lesser extent. The CYP 1 family consists of 1A1, 1A2, and 1B1 members that are capable of activating procarcinogens and high CYP1A1 activity is also associated with colorectal cancer [19]. CYP2A6 is a hepatic enzyme predominantly with some expression in specialized extrahepatic cell types. Studies about the CYP have revealed that CYP2A6 is the high-affinity metabolizer of nicotine and several procarcinogens present in cigarette smoke [20]. CYP2B is a large subfamily that encodes versatile catalysts of xenobiotics and steroid hydroxylation. CYP2B6 plays a role in the metabolism of endogenous substrates including testosterone steroid [21]. CYP2E1, an ethanol-inducible enzyme, is important in the field of toxicology and carcinogenesis, and it also has a role in drug metabolism [22]. CYP3A is the largest subfamily of CYP enzymes expressed in the liver and gastrointestinal tract. It is involved in the metabolism of 50% of therapeutic agents as well as in the activation of toxic and carcinogenic substances. As indicated in Table 1, all four compounds inhibited ECOD activity, using rat hepatic microsomes as an enzyme source. Sequoiaflavone (**15**) was the most potent inhibitor, inhibiting ECOD assay in 75.2%, by comparing the enzyme activity 24.8% with control 100%. 

Flavonoids have been reported in literature to display significant chemopreventative effects. Some classes of flavonoids, like flavones (apigenin, chrysin), flavonols (fisetin, kaempferol, morin, myrisetin, quercetin), flavanones (hesperetin and naringenin) and flavan [(±)-catechin] were studied in 7-ethoxycoumarin *O*-deethylase (ECOD) in liver microsomes from rats treated with 3-methyl-cholanthrene (MC). All the flavonoids studied inhibited microsomal ECOD activity in the following order: flavones > flavonols > flavanones, except (±)-catechin that had no effect. The double bond between C2 and C3 of the C ring, the keto group as well as the hydroxyl groups in the C5 and C7 positions of A ring in the flavones and the hydroxyl group in the C3 position of C ring in the flavonol classes, respectively, were important factors for the inhibition of the enzyme [23].

Sequoiaflavone is a flavone dimer and has a double bond between C2 and C3 of the C ring and hydroxyl groups in the C5 and C7 positions of a ring. Inhibition of CYPs enzymatic activity by biflavonoids has not been cited in the literature. Most studies report the inhibition of CYP1-catalyzed by 7-ethoxyresorufin O-deethylation (EROD) activity [24].

The isoflavones **9**, **10** and **11** inhibited ECOD activity in the following order: piscigenin (**11**) > 5,4′-OH-7,3′,5′-OMe-isoflavone (**9**) > 7,3′-di-O-methylorobol (**10**). Substitution of a methoxy group by a hydroxy group at C-7 has a most pronounced effect in reducing of ECOD activity than the absence of substituent at the C-5′ in isoflavone **10**.

Flavonoids with multiple hydroxyl groups have been documented as effective CYP1 inhibitors, whereas little is known regarding the inhibitory activities of polymethoxylated flavonoids on CYP1 enzymes. Recent studies suggest that CYP1A1 and CYP1B1 enzymes show substrate specificity for flavonoids with multiple methoxy groups [25].

Inhibition of CYP1 enzymatic activity has been reported for genistein, a prominent isoflavone with tumor suppressing activity, found primarily in nuts and soy-based products. Genistein demonstrated effective inhibition of CYP1 enzyme activity by EROD and the DMBA-induced DNA damage assay [26]. Biochanin A, the 4′-methoxylated derivative of genistein, was a more effective inhibitor of CYP1A1 and CYP1B1 enzymatic activities, as it was earlier noted for the flavone acacetin [26]. Substitution of a methoxy group at the 4′-position enhances the inhibition of the isoflavones towards the CYP1A1 and CYP1B1 enzymes [27].

5,7-Dimethoxyflavone (DMF) isolated from a Malaysian Piper species, reduced CYP1A1 EROD activity of the HepG2 cell virtually down to zero [28]. It is remarkable that two so seemingly similar compounds as DMF and chysin can have such different effects, *i.e.*, the 5,7-dimethoxy compound is a potent inhibitor of CYP1A1 protein whereas the 5,7-dihydroxy compound is a potent inducer [28]. Thus, methylation of flavonoids seems to be an important feature determining enzyme inhibitory properties [25].

Natural products have been in use since ancient times as medicines and spices, and the use of herbal remedies and dietary supplements is ever increasing. It is now well established that plant chemicals can affect or modulate drug-metabolizing enzymes [29]. With this resurgence of interest in natural products, focus on the interaction of the latter with xenobiotic-metabolising enzymes has received increased attention. Many flavonoids are reported to interact with phase II-enzymes like GST, increasing or reducing its activity. Flavonoids such as daidzein, genistein, morin and silymarim have been report to possess GST activity inductor effects [4]. Other flavonoids isolated from plant species (*Croton sp.*, *Curcuma sp.*, *Eurycoma sp.*, *Orthosiphon sp.* and *Cinnamomum sp*.) have inhibitory effects on glutathione *S*-transferases in blood platelets as well as in cancer cell lines [30]. During treatment of many cancers, there is often development of drug resistance in a tumour that was originally sensitive to treatment resulting in a phenomenon known as multidrug resistance (MDR). Many mechanisms are involved in MDR and these include alterations in drug transport resulting in impaired entry or enhanced efflux of the drug from the tumour cell, enhanced DNA repair, alterations in target proteins and alterations in drug metabolism [31]. One of the most important detoxifying enzymes that is involved in MDR belongs to GST family.

In this report, the effect of *Ouratea ferruginea* flavonoids toward *in vitro* GST activity was evaluated in the hepatic cytosolic fraction of rat. Our study has revealed that the compounds inhibited rat GST activity *in vitro*. Piscigenin (**11**) and 5,4′-dihydroxy-7,3′,5′-trimethoxyisoflavone (**9**) showed the best inhibitory effects, inhibiting almost 70 and 75% of GST activity, respectively.

The inhibitory effect of 7,3′-di-*O*-methylorobol (**10**) on GST activity was not observed. GSTs are promising therapeutic targets because speciﬁc isozymes are overexpressed in a wide variety of tumours [32].

Considering the structure activities relationships of the tested isoflavones, the observed effect of **9**, **10**, and **11** (Table 1), allow us to propose that the ring B of 4′-hydroxy-3′,5′-dimethoxyisoflavone made a difference in the GST enzyme inhibition. It may also be observed that the presence of a hydroxyl or methoxyl group in position C-7 causes a similar effect in the inhibition of GST enzyme as can be seen in Table 1 comparing isoflavones **9** and **11**. However, the absence of a methoxy group at C-5′ has a significant effect for the GST as observed with compounds **9** and **10**.

## 3. Experimental

### 3.1. Instrumentation and Reagents

CD spectra and IR were recorded on a JASCO 815 and Vertex-70 spectrometer, respectively. ^1^H-NMR spectra, (400.0 and 500.0 MHz) and ^13^C-NMR spectra (100.0 and 125.0 MHz) were recorded on Bruker AC 400 and 500 spectrometers using DMSO-d_6_, D_3_COD or CDCl_3_ with TMS as internal standard. GC-MS mass spectrometer Shimadzu QP2010 Plus by electron impact ionization (70 eV). LC-EM-IES spectra, Shimadzu LC-EM-TOF (225-07100-34) by electron-spray ionization. HPLC Shimadzu LC-DAD, diode array detector, C-18 column (250 mm × 4,6 mm × 5 μm, Shimadzu) and HPLC Shimadzu LC-20AV connected to a detector UV-Vis (254 nm), C-18 column (250 mm × 10 mm × 5 μm, Supelco) were used for analytical and preparative purposes, respectively. Column chromatography with silica gel (Vetec and Aldrich 0.05–0.20 mm) and Sephadex LH-20 (Sigma); for silica gel TLC plates w/UV254, aluminum backed (Merck and Sorbent) were used to analyze the fractions collected from column chromatography (CC) that were revealed by visualization under UV (254 and 366 nm), AlCl_3_-ETOH (1%), Lieberman-Burchard and/or Godin reagents or exposure to iodine vapor. All the GST and CYP inhibition assays were purchased from Sigma-Aldrich (St. Louis, MO, USA). The standard samples used in HPLC were agathisflavone, 7′′-methylagathisflavone, amenthoflavone, podocarpusflavone and luxenchalcone previously isolated from *Ouratea* and *Luxemburgia* genus [8] and syringic acid (Sigma-Aldrich).

### 3.2. Plant Material

The leaves and stems of *Ouratea ferruginea* Engl. (Ochnaceae) were collected in the campus of Embrapa in Belém, Pará State, Brazil. The species was identified by one of the authors (S. T. Rodrigues), a voucher specimen (Nº IAN-183954) was deposited at the herbarium of Embrapa Amazônia Oriental, Belém, Pará, Brazil. 

### 3.3. Extraction and Isolation

The dried and powdered leaves (968.57 g) and stems (3645.97 g) were extracted initially with 4 L *n*-hexane (leaves) and 12 L dichloromethane (stems), and both with methanol (4 L leaves and 12 L stem), five times at room temperature by three days each extraction. The solutions of leaves (L) and stems (S) were concentrated under vacuum to yield the *n*-hexane (H) and methanolic (M) extracts from the leaves (OFLH: 18.95 g and OFLM: 152.26 g) and dichloromethane (D) and methanolic (M) extracts from the stems (OFSD: 15.33 g and OFSM: 197.98 g). Part of the methanol extract from the leaves (OFLM, 85.67 g) and from stems (OFSM, 56.56 g) were suspended on MeOH:H_2_O (8:2) and successively extracted with *n*-hexane, CH_2_Cl_2_, and EtOAc yielding the fractions from the leaves (OFLMFH: 1.49 g, OFLMFD: 2.31 g and OFLMFA: 6.97 g, respectively, and the residue OFLMR: 66.59 g) and fractions from the stems extract (OFSMFH: 0.45 g, OFSMFD: 2.75 g, OFSMFA: 2.43 g, respectively, and the residue OFSMR: 50.23 g). These were fractionated on silica gel and/or Sephadex LH-20 columns using adequate eluents. The OFLH fraction (5.0 g) was chromatographed on a silica gel column, eluted with hexane and increasing the polarity with CHCl_3_ and EtOAc. Forty-one fractions were collected. Subfractions 15–16 afforded a mixture of friedelin and friedelinol (**1** + **2**; 55.0 mg). The fraction OFLMFH (1.49 g) was chromatographed on a silica gel column eluted with hexane and increasing the polarity with CHCl_3_ and EtOAc. Twenty-five fractions were collected and analyzed by TLC. Subfraction 8 furnished a mixture of friedelin and lupeone (**1** + **3**; 8,5 mg) and subfraction 16 afforded a mixture of sitosterol, stigmasterol and campesterol (**4** + **5** + **6**; 14.0 mg).The components of each mixture were confirmed by GC-MS analysis.

The fraction OFLMFD (2.31 g) was fractionated by CC on silica gel using mixtures of hexane, dichloromethane, EtOAc and MeOH, increasing gradually the polarity, as eluents. Fifteen subfractions were collected. The subfractions 26–29 were analyzed by ^1^H-NMR and characteristic signals of a mixture of biflavonoids were identified. The subfractions 26–29 (15 mg) were further analyzed by HPLC [(water-acetic acid 1%): methanol-acetonitrile (32:61:7), at a flow rate of 1 mL·min^−1^, injection volume of 10 μL, 254 nm] and compared with standard samples previously isolated from other Ochnaceae species. HPLC [C_18_ preparative column, water-methanol-acetonitrile (25:68:7, isocratic mode), 5 mL·min^−1^ flow rate, 254 nm] was used to isolate amenthoflavone (Rt 8.096, **14**, 7 mg), syringic acid (Rt 3.294, **22**, 2.0 mg), and sequoiaflavone or 7-*O*-methylamenthoflavone (Rt 22.334, **15**, 3.0 mg).

Part of the OFLMFA fraction (2.50 g) was chromatographed on a silica gel column eluted with CHCl_3_ and increasing the polarity with MeOH. Twenty four fractions were collected and analyzed by TLC. Fraction 10–11 (630 mg) was chromatographed by the same method described above and twelve subfractions were collected. Subfraction 6–9 (169.0 mg) was filtered on Sephadex LH-20 and yielded 2*R*,3*R*-epicatechin (**13**, 13 mg) which absolute configuration was defined by circular dichroism spectrum analysis (500–200 nm; 22 °C). Substance **13** (2.0 mg) was treated with Ac_2_O/pyridine (1:1) to yield the peracetyl derivative **13a.**

Fraction OFSD (10.0 g) was chromatographed on a silica gel column eluted with hexane and increasing the polarity with CHCl_3_, EtOAc and methanol. Ninety-seven fractions were collected. Subfraction 6 yielded friedelin **(1**; 82.0 mg), and 7–8 yielded a mixture of friedelin and friedelinol (**1** + **2**; 126.0 mg). Subfractions 13-15 afforded a mixture of sitosterol, stigmasterol and campesterol (**4** + **5** + **6**; 297.0 mg). The group of subfractions 21–25 were purified by preparative TLC using hexane/AcOEt (1:1) to yield the compound 5,4′-dihydroxy-7,5′,3′-trimethoxyisoflavone (**9**; 3.3 mg**)**, subfractions 27–28 were purified by preparative TLC using the same conditions and furnished 7,3′-di-O-methylorobol (**10**; 6.0 mg**)**. The other fractions of preparative TLC 27-28-p2, 27-28-p3 and 27-28-p4 were identified syringic aldehyde (**16**; 2.0 mg); 2,6-dimethoxybenzoquinone (**23**; 3,0 mg) and a mixture of 2,6-dimethoxybenzoquinone and 2,6-dimethoxyhydroquinone (**23** + **17**; 2.0 mg), respectively. Subfractions 33–34 were crystallized from petroleum ether to give ferulic aldehyde crystals (**18**; 1,9 mg) and the mixture of 5-hydroxy-7,3′,4′,5′-tetramethoxyisoflavone and 5,4′-dihydroxy-7,5′,3′-trimethoxyisoflavone (**12** + **9**; 4,9 mg) was present in the supernatant. Ferulic aldehyde **18** (1.9 mg) was treated with diazomethane to yield **18a** derivative. Subfraction 50–51 was crystallized from chloroform to give piscigenin (**11**; 5.0 mg). Subfraction 68–69 furnished a residue insoluble in chloroform, that was treated with Ac_2_O-pyridine (1:1) and identified as a mixture of sitosterol and stigmasterol glycosides acetate derivatives (**7** + **8**; 38.0 mg).

The fractions OFSMFH (0.45 g) and OFSMFD (2.52 g) were chromatographed on a silica gel column, eluted with hexane and increasing the polarity with CH_2_Cl_2_, CHCl_3_ and methanol, twenty-two subfractions were collected. Subfraction 6 was purified by preparative TLC using CH_2_Cl_2_ to afford the mixture of sitosterol, stigmasterol and campesterol (**4** + **5** + **6**; 20 mg); subfraction 14 afforded the mixture of vanillic acid, 1-hydroxy-2-methoxy-4-(1*E*-3-hydroxypropenyl)benzene and 3,5-dimethoxy-4-hydroxy-dihydrocinamaldehyde (**19** + **20** + **21**; 3,0 mg).

The structures of the compounds were determined by IR, ^1^H- and ^13^C-NMR (1D an 2D techniques), mass spectrometry including GC-MS and HPLC-MS analysis of natural compounds and some derivatives.

*5-Hydroxy-7,3*′*,4*′*,5*′*-tetramethoxyisoflavone* (**12**). Pale yellow crystals; ^1^H-NMR (400 MHz, CDCl_3_) δ: 3.87 (*s*, 6H), 3.89 (*s*, 6H), 6.39 (*d*, 1H, *J* *=* 2.5 Hz), 6.41 (*d*, 1H, *J* = 2.5 Hz), 6.72 (*s*, 2H), 7.89 (*s*, 1H), 12.84 (*s*, 1H); ^13^C-NMR (100 MHz, CDCl_3_) δ: 55.9, 56.3, 60.9, 92.2, 98.0, 105.3, 106.0, 123.7, 126.0, 138.0, 152.7, 153.0, 157.5, 162.4, 165.3, 180.3.

*5,4*′*-Dihydroxy-7,3*′*,5*′*-trimethoxyisoflavone* (**9**). Pale yellow crystals; ^1^H-NMR (400 MHz, CDCl_3_) δ: 3,90 (*s*, 3H), 3.95 (*s*, 6H), 5,62 (*s*, 1H), 6.41 (*d*, 1H, *J* *=* 2.1 Hz), 6.43 (*d*, 1H, *J* = 2.1 Hz), 6.77 (*s*, 2H), 7.90 (*s*, 1H) 12.86 (*s*, 1H), ^13^C-NMR (100 MHz, CDCl_3_) δ: 55.7, 56.4, 92.3, 98.1, 105.6, 105.8, 124.0, 135.3, 146.9, 152.6, 158.0, 162.9, 165.4, 180.9.

### 3.4. Preparation of Liver Fractions

Male Wistar rats (130 ± 150 g weight) were obtained from Universidade da Zona Oeste (UEZO). The animals were kept under light- and temperature-controlled conditions and were provided with rodent laboratory chow and water *ad libitum*. The rats were euthanized prior to the preparation of cytosolic and microsomal fractions. The livers were removed, placed in ice-cold 0.1 M phosphate buffer (pH 7.4) and then homogenized. Fractions were prepared by differential centrifugation [33] and protein content was determined by Peterson’s methods [34]. 

### 3.5. Preparation of Samples

5,4′-Dihydroxy-7,3′5′-trimethoxyisoflavone (**9**), 7,3′-di-*O*-methylorobol (**10**), piscigenin (**11**), and sequoiaflavone (**15**) were accurately weighed. Solutions were prepared at a concentration of 8 mg/mL using DMSO as a solvent. CYP1A and GST assays were used aliquots of 10 µL of solution.

### 3.6. CYP Inhibition Assays

Ethoxycoumarin *O*-desalkylation by rat liver microsomes was used as a probe to determine CYP1A activity. Ethoxycumarin (1 mM), NADPH (1 mM), magnesium chloride (10 mM) and rat liver microsomes (5.5 μg) were incubated in phosphate buffer (0.1 M), pH 7.8 (0.5 mL ﬁnal volume). Incubations were at 37 °C for 5 min. The reaction was terminated with trichloroacetic acid (TCA) 2.5% followed by centrifugation (1000 g, 10 min). Immediately before ﬂuorescence determination, 2 mL of glycine-NaOH buffer were added and fluorescence of cumarin was determined (excitation: 390 nm and emission 440 nm). CYP activity was calculated as amount of produced cumarin per minute per protein mg. Assays containing phytochemicals were compared with control with DMSO (dimethylsulfoxide).

### 3.7. GST Inhibition Assays

The conjugation of glutathione to 1-chloro-2,4,-dinitrobenzene (CDNB) was measured as a non-speciﬁc substrate for GST activity [35]. To a quartz cuvette reduced cytosolic fraction (10 μg), CDNB (2 mM) and 100 mM potassium phosphate buffer (pH 6.1) were added in a ﬁnal volume of 1 mL. The cuvette was placed in a spectrophotometer and the reaction initiated by the addition of glutathione (5 mM). The increase in absorbance at 340 nm over a 3 min period was measured at room temperature. An extinction coefﬁcient of 9.6 cm/mM was used to determine activity from the initial slope of the reaction. GST activity was calculated as amount of produced conjugated per minute per protein mg. Assays containing phytochemicals were compared with control with DMSO (dimethylsulfoxide).

## 4. Conclusions

The results obtained in this work allowed us to expand the knowledge about the chemical composition of the genus *Ouratea* and the Ochnaceae family in particular. This is first chemical study of the species *Ouratea ferruginea*. In this work 24 substances were identified in the extracts of stems and leaves. The substance 7,3′-dimethylorobol (**10**) is being registered for the first time in Ochnaceae. No record was found in the literature of the substance 5-hydroxy-7,3′,4′,5′-tetramethoxyisoflavone (**12**). The compounds 5,4′-dihydroxy-7,3′,5′-trimethoxyisoflavone (**9**) and piscigenin (**11**), have not been previously identified in *Ouratea*. The presence of biflavonoids such as amentoflavone (**14**) in the extracts of leaves confirms the botanical classification, since they are quimiossistematic markers of the genus. The stems of *Ouratea ferruginea* is a source of methoxyl isoflavones derivatives. 

In this report, we also describe the ECOD and GST inhibitory assays. All the tested substances in inhibition assays showed inhibitory effects against at least in one of the enzymes used (ECOD and GST). The moderate and strong inhibitory effects are relevant, because little is known about the enzymatic inhibition effects of methoxyisoflavones and biflavonoids.
